# Identification of an unusual cluster of human granulocytic anaplasmosis in the Estrie region, Québec, Canada in 2021

**DOI:** 10.14745/ccdr.v48i05a02

**Published:** 2022-05-05

**Authors:** Laurence Campeau, Valérie Roy, Geneviève Petit, Geneviève Baron, Jacinthe Blouin, Alex Carignan

**Affiliations:** 1 Canadian Field Epidemiology Program, Public Health Agency of Canada, Ottawa, ON; 2 Department of Microbiology and Infectious Diseases, Université de Sherbrooke, Sherbrooke, QC; 3 Direction de la santé publique de l’Estrie (Estrie Public Health Department), Sherbrooke, QC; 4 Department of Community Health Sciences, Université de Sherbrooke, Sherbrooke, QC

**Keywords:** **:** *Anaplasma phagocytophilum*, human granulocytic anaplasmosis, tick-borne disease, zoonosis

## Abstract

**Background:**

Human granulocytic anaplasmosis (HGA) is a potentially severe tick-borne infection caused by the bacterium *Anaplasma phagocytophilum* (*A. phagocytophilum*) of the genus Rickettsia. Here, we describe the epidemiological and clinical characteristics of an unusual cluster of HGA cases detected in the Estrie region in Québec, Canada, during the 2021 transmission season.

**Methods:**

Confirmed cases of HGA were defined as individuals with typical clinical manifestations and a positive polymerase chain reaction assay. The cases were interviewed using a structured questionnaire and clinical data was obtained from medical records.

**Results:**

A total of 25 confirmed cases were identified during the 2021 transmission season, thus constituting the largest known cluster of HGA in Canada. The most common symptoms reported were fever, fatigue and headaches. Laboratory investigations found that 20 (80%) of the patients had thrombocytopenia and 18 (72%) had leukopenia at presentation. Almost half of the patients required hospitalization (n=11, 44%), with a median duration of four days (interquartile range [IQR] 2.5–5 days), including one patient who required intensive care. No deaths were recorded during the study. Epidemiological investigation found that all cases were domestically acquired, and yard maintenance was the most prevalent at-risk activity identified. Only seven (28%) cases had been aware of a tick bite in the previous two weeks.

**Conclusion:**

Detection of this unusual cluster of HGA cases provides further evidence that *A. phagocytophilum* may now be established along the southern border of Québec. Clinicians should consider HGA when assessing patients with typical symptoms and recent exposure to high-risk environments for tick bite.

## Introduction

Human granulocytic anaplasmosis (HGA) is a tick-borne infection caused by the bacterium *Anaplasma phagocytophilum* (*A. phagocytophilum)* of the genus Rickettsia. In Northeastern America, the main vector of the disease is *Ixodes scapularis* ((1)), commonly known as the blacklegged tick, that also transmits *Borrelia burgdorferi*, the causative agent for Lyme disease (LD). Individuals usually develop nonspecific symptoms such as fever, chills, myalgias, malaise, severe headaches and gastrointestinal symptoms one to two weeks after exposure ((2)). While the illness can be severe and possibly life threatening if left untreated, antimicrobial treatment generally leads to resolution of symptoms within 48 hours ((3)).Human granulocytic anaplasmosis is primarily endemic in the upper Midwestern and Northeastern United States ((4)), but *A. phagocytophilum* has been detected in tick populations of all Canadian provinces in recent years. Nonetheless, data on HGA infections among humans in the Canadian context are limited because Manitoba and Québec are the only provinces where HGA is a reportable disease. Between 2015 and 2019, 37 confirmed cases were reported in Manitoba ((5)). In Québec, three confirmed cases have been reported to public health since the disease became subject to mandatory reporting for laboratories in 2019. This included one case in the Estrie region, which is located along the southern border of eastern Québec (*personal communication, Institut national de santé publique du Québec [INSPQ]*). Here, we describe the epidemiological and clinical characteristics of an unusual cluster of HGA infections reported in the Estrie region during the 2021 transmission season.

## Methods

### Study setting, population, and design

We conducted a retrospective case series analysis in the Estrie region, Québec, Canada that has a total population of 489,479 ((6)). This region accounts for the majority of LD cases in the province and shares its southern border with three of the eight states in the United States with the highest incidence of HGA: Vermont; New Hampshire; and Maine ((4)). Our study sample included all confirmed cases of anaplasmosis in this region from May 1, 2021, to November 20, 2021. A confirmed HGA case was defined as an individual with typical clinical manifestations and a positive polymerase chain reaction (PCR) assay ((7)). As anaplasmosis is a notifiable disease in Québec, the list of patients with positive PCR results was extracted from the regional notifiable diseases database of the *Direction de la santé publique de l’Estrie*.

### Laboratory methods for detection of tick-borne infections

All the diagnostic and confirmatory microbiological tests for *Anaplasma phagocytophilum* and other potential coinfections were performed either at the National Microbiology Laboratory in Winnipeg, Manitoba, at the *Laboratoire de santé publique du Québec* in Sainte-Anne de Bellevue, Québec, or at the National Reference Center for Parasitology in Montréal, Québec. Detailed laboratory testing methods are available in **Annex**.

### Data collection

One infectious disease fellow and one field epidemiologist in collaboration with the Communicable Disease team at the *Direction de la santé publique de l’Estrie*, performed chart reviews in three different acute care hospitals within the *Centre intégré universitaire de santé et service sociaux de l’Estrie – Centre hospitalier universitaire de Sherbrooke (CIUSSS de l’Estrie – CHUS)*, where the confirmed cases were evaluated and treated. A standardized data abstraction form, which was developed by our research team after an initial literature review and pre-tested on one patient, was used for data collection. Past medical history was collected to calculate the Charlson Comorbidity Index ((8)), along with demographic, microbiological, and treatment data. Data on symptoms, clinical signs, and laboratory findings were also collected. A standardized questionnaire to assess history of tick bite and possible exposure sources, including the location, the activities undertaken and their frequency, was built and all patients underwent a phone interview. Activities undertaken by the cases in the two weeks prior to symptom onset were considered at-risk if they took place in an area known to be endemic for LD and the environment was suitable for ticks (e.g. grassy, brushy or wooded areas). If a case practiced multiple at-risk activities during the time period, all activities were included in the descriptive analysis.

### Geographic information and data visualization

Spatial data was uploaded to a geographic information software (QGIS 3.10.9) to develop a map of the location of residence of cases.

### Statistical analysis

Data cleaning and descriptive analyses were performed using Excel 2016 and Stata version 15.1 (StataCorp, College Station, Texas, United States).

### Ethics approval

The *Comité d'éthique clinique et organisationnelle* (institutional review board) of the CIUSSS de l’Estrie-CHUS approved this study (Project #2022-4465).

## Results

**Figure 1:**
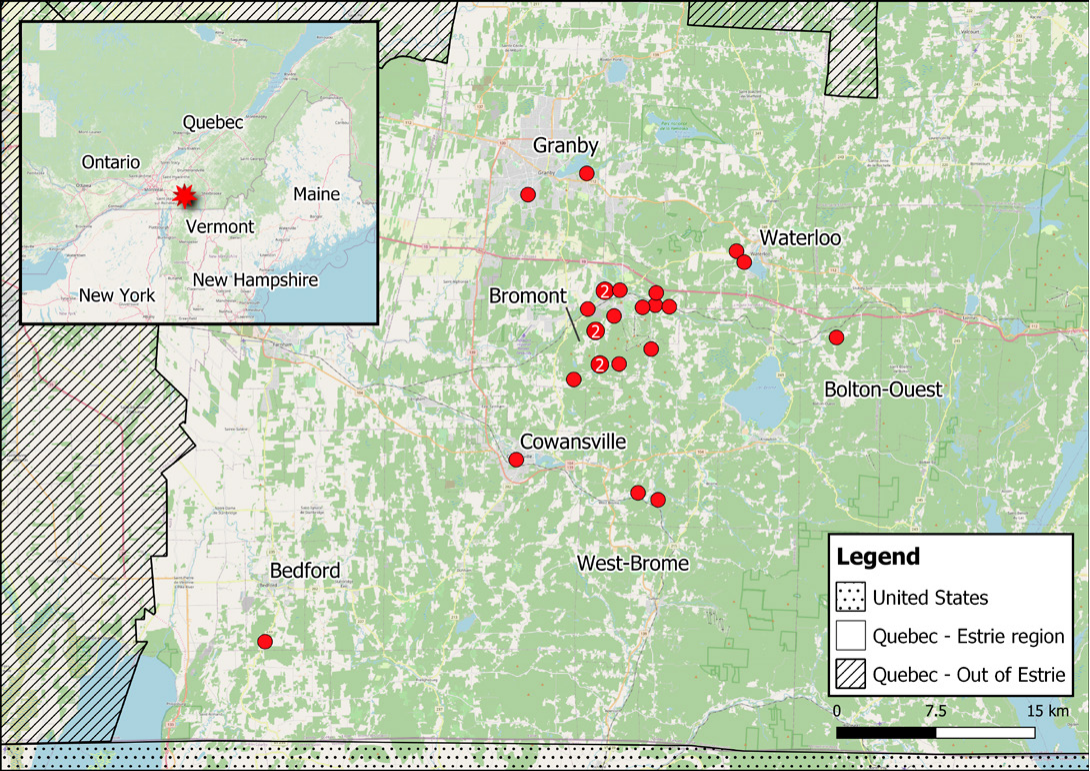
Location of residence of confirmed cases of human granulocytic anaplasmosis in the Estrie region^a^, 2021 ^a^ Map represents the Estrie region of Québec. The location of residence for confirmed cases of human granulocytic anaplasmosis is represented by red dots. The red dots indicate that 16 cases are located in proximity to the town of Bromont. The other municipalities included in the map are Bedford, Bolton-Ouest, Cowansville, Granby, Waterloo and West-Brome, with one or two confirmed cases residing in each

During the study period, 25 confirmed cases were identified in the Estrie region (**Figure 1**). The patients’ demographic and clinical characteristics are summarized in **Table 1**. The majority of cases were male (n=15, 60%) and the median age was 65 years. All cases were either permanent or seasonal residents of the regions of La Pommeraie or Haute-Yamaska at the time of exposure, with a majority of cases residing in the town of Bromont (n=16, 64%). None of the cases reported out-of-province travel in the previous two months. The activity most often reported by cases was yard maintenance (n=22, 88%), which included gardening, lawn mowing and wood chopping. Additionally, 48% (n=12) of the cases reported outdoor recreational activities such as walking, mountain biking and shooting practice, whereas five (20%) cases reported potential exposure while taking care of farm animals or visiting a farm. Overall, 28% (n=7) of the cases had observed a tick attached to their skin in the two weeks prior to symptom onset.

**Table 1:** Characteristics of confirmed human granulocytic anaplasmosis cases

Characteristic	n=25	
	n	%
Sex		
Female	10	40%
Male	15	60%
Age		
Years, median (IQR)	65	53–70
Municipality of residence		
Bedford	1	4%
West Bolton	1	4%
Bromont	16	64%
Cowansville	1	4%
Granby	2	8%
Waterloo	2	8%
West Brome	2	8%
At-risk activities reported^a^		
Yard maintenance	22	88%
Outdoor recreational activity	12	48%
Farm visit or animal care	5	20%
Recent out-of-province travel	0	0%
Tick bite ≤2 weeks preceding symptom onset	7	28%
Charlson comorbidity index		
0	21	84%
1	2	8%
≥2	2	8%
Symptoms and clinical signs		
Fever^b^	25	100%
Duration of fever in days (median [IQR])^c^	4	2–5
Sweating	17	68%
Fatigue	24	96%
Myalgia	20	80%
Arthralgia	12	48%
Vomiting	11	44%
Diarrhea	9	36%
Abdominal pain	8	32%
Headache	22	88%
Cough	5	20%
Dyspnea	5	20%
Erythema migrans	0	0%
Nonspecific rash	2	8%
Outcome		
Hospitalization	11	44%
Duration of hospitalization in days (median [IQR])	4	2.5–5
Intensive care unit	1	4%
Death	0	0%
Immunosuppression^d^	3	12%

Abbreviation: IQR, interquartile range

^a^ Non-mutually exclusive categories

^b^ 23 out of 25 patients had objective fever and two had subjective sensation of fever without measurement confirmation

^c^ Data missing for two patients

^d^ Two patients were using immunosuppressing drugs (one using ustekinumab and one using prednisone) and one patient had HIV (but virologically controlled and on antiretroviral therapy)

**Figure 2:**
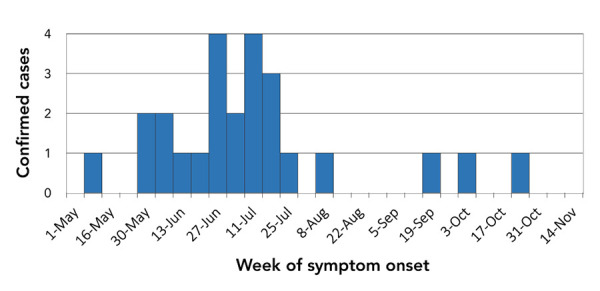
Confirmed cases of human granulocytic anaplasmosis in the Estrie region by week of symptom onset, Québec, 2021

Most patients developed symptoms in either June (n=9) or July (n=11) (**Figure 2**). All cases experienced fever and reported symptoms such as fatigue (n=24; 96%), headaches (n=22; 88%), myalgia (n=20; 80%) and sweating (n=17; 68%). A significant proportion of patients presented gastrointestinal symptoms such as vomiting (n=11; 44%), diarrhea (n=9; 36%) and abdominal pain (n=8; 32%). Two cases (8%) reported a rash; in both cases the rashes were less than 5 cm in diameter and, therefore, not characteristic of erythema migrans. The detailed hematologic and biochemical laboratory findings are listed in **Table 2**. The most frequent laboratory anomalies were leukopenia (n=18/25; 72%), thrombocytopenia (n=20/25; 80%) and mildly elevated alanine aminotransferase levels (n=14/24; 58%).

**Table 2:** Hematologic and biochemical laboratory findings

Hematologic and biochemical findings	n=25	
	Median	IQR
Leucocytes (1x10^9^/L)		
Count upon presentation	3.3	2.5–5.2
Lowest count	2.9	2.1–3.4
Neutrophiles (1x10^9^/L)		
Count upon presentation	2.4	1.4–3.9
Lowest count	1.4	0.9–1.7
Lymphocytes (1x10^9^/L)		
Count upon presentation	0.7	0.2–1.0
Lowest count	0.6	0.2–1.0
Highest count	2.5	1.5–3.7
Platelets (1x10^9^/L)		
Count upon presentation	114	72–141
Lowest count	76	61–123
Anemia^a^ (n, [%])	9	36%
Alanine aminotransferase (IU/L)		
Upon presentation	61.5	36.8–122.8
Maximum value	80	58.5–224.5
C-reactive protein (mg/L)		
Upon presentation	82	35.5–171
Maximum value	94.5	35.8–184.5
Acute kidney injury^b^	3	12%

Abbreviation: IQR, interquartile range

^a^ Anemia was defined as hemoglobin levels below 130 g/L for men and 120 g/L for women, as per local laboratory guidelines

^b^ Acute kidney injury was defined as increase of ≥1.5 × compared to baseline creatinine or increase of ≥27 mmol/L over baseline creatinine

Almost half of the patients required hospitalization (n=11; 44%), with a median duration of 4 days (interquartile range [IQR] 2.5–5 days), including one patient who required intensive care. Hospitalized patients were slightly older than those who did not require hospitalization, but this difference was not statistically significant (67.0 vs 61.3 years old; *p*=0.2). None of the patients died during the study period. All patients were treated with doxycycline for a median duration of 14 days (IQR 14–16 days).The findings of the diagnostic tests for anaplasmosis and other potential coinfections are listed in **Table 3**. *Anaplasma phagocytophilum* serology (indirect immunofluorescence assay) was performed for 21 patients during the acute phase of infection and antibodies were detected in four patients. Convalescent-phase repeated testing was performed in two patients; none showed a four-fold increase in antibody titers. Interestingly, among patients with positive serology (n=4), the time between the start of symptoms and serology was significantly longer compared to patients with negative serology (median of 18.5 days vs 4.0 days), and an indeterminate serology result was obtained in a patient whose blood was drawn seven days after symptom onset. Three patients had peripheral smears showing morulae in the neutrophils in addition to positive PCR results. For other coinfections, 11 patients out of 23 tested positive using enzyme immunoassay (EIA) for Lyme serology, of whom seven were positive for isolated line blot IgM in the confirmatory test. The other four patients were positive for Lyme western blot IgG.

**Table 3:** Results from diagnostic tests for anaplasmosis and other potential coinfections

Pathogen	Diagnostic test (positive results/total tests performed)		
	Polymerase chain reaction	Serology	Blood smear
*Anaplasma phagocytophilum*	25/25	4/21^a^	3/4^b^
*Borrelia burgdorferi*	0/1	EIA: 11/23^c^ Western blot IgG: 4/11 Line blot IgM^d^: 7/7	N/A
*Babesia microti*	0/19	0/14	0/18

Abbreviations: EIA, enzyme immunoassay; N/A, not applicable

^a^ Dilution range: 1/64-1/2048

^b^ Was found in two patients in routine blood smear

^c^ EIA positive tests were sent for western blot IgG

^d^ IgM line blot was performed only if the western blot IgG was negative

## Discussion

This report describes the epidemiological and clinical features of a cluster of HGA cases in the Estrie region, located along the southern border of Québec. A total of 25 cases have been confirmed in 2021, thus constituting the largest reported cluster of confirmed HGA cases identified during a transmission season in Canada. Since the first reported case of HGA in Canada in 2009 ((9)), surveillance data shows that HGA seroprevalence has increased among the populations of Manitoba and Ontario ((10,11)). Nonetheless, an article describing three cases in Manitoba is the only other publicly available case series that describes a cluster of confirmed HGA cases in Canada ((10)).Our data also provides further evidence that *A. phagocytophilum* may now be established in blacklegged tick populations in the Estrie region, as previously indicated by acarological surveillance programs ((12)). These findings are also consistent with a recent study that suggests an expansion of the suitable geographic areas for tick reservoirs and hosts, such as mice and deer, resulting in the emergence of tick-borne diseases in new areas ((13)). Before 2021, only three confirmed cases of human anaplasmosis had been reported to public health in Québec, including one in the Estrie region ((14)).In this study, most cases were observed in males, which is consistent with previous findings indicating that men are more likely to adopt behaviors that put them at risk of tick bites ((15,16)). Only four patients were younger than 50 years old; however, this could be partly due to an increased likelihood of asymptomatic infections among younger individuals. Yard maintenance was the most common at-risk activity reported by cases during their exposure period. This is consistent with similar finding by Porter *et al*., which found that yard work was the most common activity practiced during tick encounters in a sample of individuals who submitted ticks through a passive tick surveillance system in the Northeastern United States ((17)).Most cases had nonspecific symptoms such as fever, headaches and fatigue. Digestive symptoms were also prevalent in our case series. Laboratory abnormalities, including leukopenia, thrombocytopenia and elevated hepatic transaminase levels, were present in majority of patients. These data are consistent with the clinical and paraclinical presentations reported recently ((1,18)). The proportion of hospitalized patients seen in our sample was marginally higher than that reported in the national surveillance data in the United States from 2008 to 2012 (44% vs 31%) ((3)); however, the higher hospitalization rates were probably because HGA is not yet a well-recognized disease in our region and physicians may be less likely to identify HGA in outpatient settings.It has been reported that among patients with positive *Anaplasma phagocytophilum* serology, 4%–36% show positive serology for either *Borrelia burgdorferi* or *Babesia microti* ((1)). Interestingly, almost half of our patients had positive Lyme serology, but only two reported a nonspecific rash, which is not indicative of classic erythema migrans. Among those with positive EIA (n=11), four were positive for IgG (determined by western blot); this methodology was in line with the two-tier testing approach currently used in Canada. For those with positive EIA and negative IgG, IgM positivity was shown in all (using the line blot method). Although IgM titers are classically known to indicate a recent infection, there are limitations to the test. IgM can be falsely positive and can remain positive for months or years after the initial infection ((1)). Therefore, even if a high proportion of the patients in our case series were IgM positive, it is difficult to conclude that all patients had a coinfection, especially without the manifestation of erythema migrans. Convalescent serology would have helped the confirmation of early coinfection with LD if IgG developed afterward, but these results were not available at the time of manuscript submission. No coinfection with *Babesia microti* was diagnosed in our series; this was expected since this parasite is not commonly found in ticks in the region according to acarological surveillance programs ((12)).

### Future directions

While HGA is a nationally reportable condition in the United States ((19)), it is only reported in the provinces of Manitoba and Québec in Canada. As suggested elsewhere ((2,20)), a nationally reportable disease status would improve epidemiologic monitoring, which is especially important in identifying other newly endemic areas. Mandatory reporting would also increase physician awareness of this emerging infection, facilitating early diagnosis and treatment. Early antimicrobial treatment of HGA is critical as it reduces the risk of severe complications and may be lifesaving for individuals at higher risk of death, such as immunocompromised and elderly patients ((10)). The adoption of multiplex tests for tick-borne diseases should also be considered to facilitate the identification of emerging pathogens in areas where LD is already endemic ((21)).Improvements to current acarological surveillance strategies are also needed to preemptively identify regions where *A. phagocytophilum* is most likely to occur. This was highlighted in the conclusions of the federal framework on LD in Canada ((14)), which identified the development of a national tick-borne diseases surveillance system as a priority action item. This system would incorporate region-specific data on the distribution of vectors and the prevalence of disease-causing pathogens to improve the monitoring of the distribution of ticks capable of transmitting LD, HGA and other infections.Furthermore, the primary prevention method for tick-borne diseases, including HGA, remains the adoption of preventive behaviors that reduce the risk of tick encounters. Existing LD health promotion efforts should be reinforced and, in regions where *A. phagocytophilum* has been detected, tailored to incorporate HGA. A multi-sectoral and multidisciplinary approach that involves both human and animal health stakeholders should also be emphasized to help identify prevention strategies that leverage the One Health approach, as well as to better understand the role of tick vectors, such as deer and mice, in the emergence of new risk areas for HGA ((22)).

### Limitations of the study

Our study was limited by its observational design, as it included only cases reported to the public health department. Even if our definition of confirmed cases was based on a very specific and reliable assay (PCR), our data certainly underestimated the true burden of HGA in the region. Subclinical cases are likely to remain undetected and since this disease has only recently emerged in the area, physicians are likely to miss diagnoses due to lack of awareness. Another limitation of our study is that it was not possible to attribute the acquisition of HGA to a specific at-risk activity when multiple exposures took place in the two weeks prior to symptom onset; therefore, all activities were listed.

## Conclusion

Human granulocytic anaplasmosis is a growing public health concern in the southern regions of Québec, Canada. A reportable disease status should be considered by provincial and federal jurisdictions, and health promotion efforts that aim to reduce the risk of tick encounters should be reinforced. Clinicians should consider the possibility of HGA when assessing patients with fever, leukopenia, thrombocytopenia, elevated hepatic transaminase levels and recent exposure to high-risk environments for tick bites. The initiation of empiric treatment with doxycycline should be considered prior to reception of PCR testing results when those results cannot be obtained in a timely manner.

## Annex: Laboratory methods for detection of tick-borne infections

For *Anaplasma phagocytophilum*, the molecular detection is realized with an in-house real-time polymerase chain reaction (RT-PCR) targeting the msp2 gene. If positive, a conventional PCR and sequencing are performed to confirm and further characterize the strain of *A. phagocytophilum* ((23)). The National Microbiology Laboratory (NML) performed an indirect immunofluorescence assay (IFA) using the Focus *A. phagocytophilum* IFA IgG kit (DiaSorin Molecular, Cypress, California, United States), according to the manufacturer’s instructions ((24)).

For molecular detection of *Babesia* spp., amplification of the 18s rRNA gene common to all *Babesia* species is first performed. If positive, specific-species RT-PCR, melted-curve analysis, conventional PCR and sequencing are completed to establish species ((25)). Testing for antibody to *Babesia microti* was performed using a manual indirect immunofluorescent antibody (immunoglobulin G) assay (Imugen, Norwood, Massachusetts, United States) ((26)).

For Lyme disease, a first serological assay is performed at the *Laboratoire de santé publique du Québec* with a commercial enzyme immunoassay (ELISA Borrelia VlsE1/pepC10 IgG/IgM, Zeus Scientific, Branchburg, New Jersey, United States). If positive, the specimen is sent to the NML for confirmatory Western Blot (WB) for IgG antibodies against *Borrelia burgdorferi* (Anti-B. burgdorferi US EUROLINE IgG, Euroimmun, Lübeck, Germany). If the WB is negative, a Line Blot (LB) to detect IgM is executed (Anti-Borrelia EUROLINE-RN-AT-adv IgM, Euroimmun, Lübeck, Germany) ((27)).
